# Early Postoperative Outcomes of Phacoemulsification With Concurrent Silicone Oil Removal in a Tertiary Eye Care Hospital of Bangladesh

**DOI:** 10.7759/cureus.110408

**Published:** 2026-06-07

**Authors:** Md Rezwanul Hasan, Mominul Islam, Habiba Sultana, Nusrat Islam, Ametav Das, Md Saidur Rahman, Palash Kinda, Md Sajidul Huq

**Affiliations:** 1 Vitreo-Retina, Deep Eye Care Foundation, Rangpur, BGD; 2 Vitreo-Retina and Uvea, Ispahani Islamia Eye Institute and Hospital, Dhaka, BGD; 3 Glaucoma, Deep Eye Care Foundation, Rangpur, BGD; 4 Cornea and Anterior Segment, Deep Eye Care Foundation, Rangpur, BGD; 5 Orbit-Oculoplasty, Deep Eye Care Foundation, Rangpur, BGD; 6 Public Health, Deep Eye Care Foundation, Rangpur, BGD

**Keywords:** diabetic eye disease, phacoemulsification, retinal detachment (rd), silicone oil removal, tractional retinal detachment

## Abstract

Purpose: Silicone oil (SO) tamponade is essential in managing complex vitreoretinal conditions but is associated with complications such as cataract formation and secondary glaucoma. Combining SO removal (SOR) with cataract surgery in a single procedure may improve patient convenience and outcomes. This study aims to evaluate the early postoperative outcomes of phacoemulsification with concurrent SOR in a tertiary eye care hospital of Bangladesh.

Methods: This prospective longitudinal study was conducted at Ispahani Islamia Eye Institute and Hospital, Dhaka, Bangladesh, from January 1, 2023, to June 30, 2023. Patients who had previous surgery due to retinal detachment (RD) or advanced diabetic eye disease with SO used as an internal tamponade with postoperative cataract formation and an attached retina, confirmed by indirect ophthalmoscopic examination or B scan, depending on media clarity, were included in the study. Phacoemulsification with intraocular lens implantation was performed, followed by SOR using the 23-gauge pars plana method. Postoperative follow-ups were conducted at day 1, day 7, and at least after 1 month to record visual acuity, complications, and retinal status.

Result: Out of 42 patients, a majority of patients (13, 31.0%) belonged to the age group of 50-59 years with a mean age of 44.38 ± 14.4 years. The male-to-female ratio was 4:3. The primary pathology was tractional RD secondary to diabetic retinopathy in 24 eyes and rhegmatogenous RD in 18 eyes. There was an improvement in at least one-third of patients' visual acuity postoperatively as recorded by the Snellen chart. Retina was attached in 41 (97.6%) patients, while 1 (2.4%) patient had a redetached retina at the last follow-up.

Conclusion: Phacoemulsification with concurrent SOR is a useful method that is associated with acceptable visual and anatomical outcomes, and it eliminates the burden for another surgery for cataract extraction.

## Introduction

Silicone oil (SO) tamponade is an essential tool in managing complex vitreoretinal pathologies, including severe cases of retinal detachment such as giant retinal tears, trauma, proliferative vitreoretinopathy (PVR), and diabetic tractional retinal detachment (TRD). The unique properties of SO allow it to maintain retinal flattening and preserve intraocular pressure (IOP), which are critical in preventing the recurrence of retinal detachment and in ensuring the structural integrity of the eye following surgery. Unlike other tamponade agents such as gas or air, SO provides a clear medium through which retinal examination can be performed postoperatively, making it a preferred choice in cases where close postoperative monitoring of the retina is necessary [1].

Despite its benefits, the use of SO is not without complications. The most significant of these include SO emulsification, secondary glaucoma, cataract formation, and corneal band keratopathy [2]. Emulsification of SO can occur due to several factors, including the mechanical energy imparted by intraocular instruments during surgery, which disrupts the oil's structure, leading to the formation of smaller oil droplets that can migrate within the eye and cause further complications [3]. Moreover, prolonged presence of SO within the eye increases the likelihood of cataract formation and secondary glaucoma. It has been observed that the longer SO remains in the eye, the higher the risk of developing these complications [4]. Even short durations of SO tamponade have been associated with the development of cataracts, indicating that the oil's mechanical impact on the lens may play a more significant role in cataractogenesis than its potential toxic effects [5,6].

The management of patients who have undergone SO tamponade often necessitates a combined surgical approach, particularly when cataract surgery is indicated. SO removal (SOR) can be challenging, especially given the risk of retinal redetachment that accompanies the procedure [7]. In such cases, combining cataract surgery with SOR in a single operative session can reduce overall surgical time and potentially minimize the risk of complications [8,9]. However, the timing of SOR remains a topic of debate among ophthalmologists. While some advocate for early removal to reduce the risk of complications, others suggest that delaying the procedure may be beneficial in certain cases, allowing for more stable retinal conditions [10].

The incidence of retinal redetachment following SOR varies widely in the literature, with reported rates ranging from 0% to 32% [10,11]. This variation is likely due to differences in study designs, sample sizes, the duration of follow-up, and the underlying retinal conditions being treated. The complexity of the disease process and the severity of retinal detachment also significantly influence the likelihood of redetachment post-SOR [2]. In addition, the scarcity of comprehensive studies exploring strategies for SOR, rates of complications, and associated risk-benefit ratios underscores the need for further research in this area [9].

In the context of this prospective study, we aim to explore early postoperative outcomes, including anatomical and visual outcomes of patients undergoing phacoemulsification with concurrent SOR in a tertiary eye care setting in Bangladesh. By examining the associated complications and demographic factors, this study seeks to contribute to the limited body of knowledge on the subject and provide insights that could inform clinical practice and improve patient outcomes.

## Materials and methods

The present study employed a prospective cohort design to investigate the early postoperative outcomes of phacoemulsification with concurrent SOR in patients who had previously undergone vitrectomy with SO tamponade, resulting in subsequent cataract formation. Conducted at the Department of Vitreo-Retina, Ispahani Islamia Eye Institute and Hospital in Dhaka, Bangladesh, the study spanned a duration of six months, from January 1st, 2023, to June 30th, 2023.

The study population consisted of patients meeting specific inclusion criteria: those with a history of vitrectomy where SO was used as an internal tamponade and who subsequently developed cataracts. Inclusion criteria specified patients with rhegmatogenous retinal detachment (RRD), TRD, or combined retinal detachment. Patients were excluded if they had undergone vitreo-retinal surgery with tamponading agents other than SO, had significant ocular pathologies such as coloboma or macular pathology, were aphakic or pseudophakic, had developed secondary glaucoma due to SO, had corneal complications such as band keratopathy, or if their clinical records were incomplete.

Purposive sampling was employed to select eligible patients from the clinical records of the Vitreo-Retina department. The sample size was determined based on the expected proportion of patients undergoing the combined procedure, set at 90%, with a precision level of 10% of that proportion. This calculation yielded a required sample size of 42 patients.

Purposive sampling was employed as this study aimed to evaluate outcomes of a specific surgical procedure in a well-defined patient population with clearly established inclusion criteria. This non-probability sampling method was appropriate given the study's exploratory nature and the need to recruit patients with the precise combination of clinical characteristics: prior vitrectomy with SO tamponade, subsequent cataract formation, and confirmed attached retina. While purposive sampling limits generalizability, it ensures that all enrolled patients meet the strict eligibility criteria necessary to answer the clinical question. Similar sampling strategies have been employed in published studies of combined phacoemulsification and SOR.

Preoperative axial length measurement and IOL power calculation were performed using immersion A-scan ultrasonography in all cases because optical biometry was not feasible due to dense cataract or media opacity from SO. The SRK/T formula was used to target emmetropia or slight myopia (−0.25 to −0.50 D), depending on the fellow eye status. A constant was adjusted according to the presence of SO (typically adding +0.5 to +1.0 D or per the manufacturer's recommendation for the specific oil viscosity).

All surgical procedures were performed by a single experienced vitreoretinal surgeon (MRH) under peribulbar anesthesia. A 2.2 mm temporal corneal incision was made for phacoemulsification, followed by a continuous curvilinear capsulorhexis. Hydrodissection was performed to separate the lens from the capsule, and phacoemulsification was performed to fragment the nucleus. Automated irrigation/aspiration was then used to remove the cortical material, and a foldable intraocular lens (IOL) was implanted into the capsular bag. The SOR was performed through a 3-port pars plana vitrectomy (PPV) approach. Three 23-gauge sutureless entry ports were created: one inferotemporal, one superior, and one inferior to facilitate complete access to the vitreous cavity. SO was aspirated using a supratemporal approach with a vacuum setting of 480-650 mmHg. The fluid-air exchange technique was used to help expel any remaining SO, and retinal integrity was confirmed before removing the trocars. The postoperative inspection was performed to ensure no leakage from the sclerotomy sites; if any leakage was detected, the sclerotomies were sutured with 7-0 Vicryl. A detailed record was kept of any intraoperative complications during phacoemulsification, particularly posterior capsular rupture. Lens touch or zonular stress during the preceding primary vitrectomy with SO insertion was noted from prior operative records when available, as this may have contributed to accelerated cataract formation.

Data collection involved a meticulous review of patient case notes and clinical records. Specifically, preoperative and postoperative visual acuity measurements were recorded. Additionally, any documented postsurgical complications were meticulously noted. Data were entered into Microsoft Excel (Microsoft Corp., Redmond, WA, USA) and analyzed using SPSS version 25 (IBM Corp., Armonk, NY). Descriptive statistics were used to summarize demographic and clinical characteristics. Categorical variables were presented as frequencies and percentages.

Visual acuity was classified according to the International Classification of Diseases-11 (ICD-11) (2018) criteria for distance vision impairment: mild impairment: <6/12 to ≥6/18, moderate impairment: <6/18 to ≥6/60, severe impairment: <6/60 to ≥3/60, and blindness: <3/60 (including light perception and no light perception). For inferential analysis, preoperative and postoperative visual acuity were compared using the Wilcoxon signed-rank test. This non-parametric test was selected because: (a) the data are paired (same patients measured twice), (b) the visual acuity categories are ordinal, and (c) the data are not normally distributed. A p-value < 0.05 was considered statistically significant. Additionally, McNemar's test was used to compare the proportion of patients with blindness (visual acuity: <3/60) before and after surgery. This test is appropriate for paired dichotomous data.

The study adhered strictly to the ethical principles outlined in the Helsinki Declaration. Informed written consent was obtained from all participants, ensuring their understanding and voluntary participation. Confidentiality of patient information was maintained throughout the study process, with no identifiable personal data included in the analysis or publication of results. Measures were in place to avoid any form of deception, ensuring the integrity of the study and the well-being of participating patients. The study was approved by the ethical institutional review board of Ispahani Islamia Eye Institute and Hospital, Dhaka, Bangladesh (Ref No: IIEI&H/ED/36/0/2023).

## Results

Demographic characteristics

The study comprised a total of 42 respondents. The gender distribution highlighted a slightly higher participation of males, accounting for 57.1% (n=24) males and 42.9% (n=18) females. The age group with the highest representation was 50-59 years, making up 31.0% (n=13) of the respondents. This was followed by the 40-49 years age group, which constituted 23.8% (n=10) of the respondents. The mean age of respondents was 44.38 ± 14.4 years, with the youngest being 16 years old and the oldest 67 years old.

Regarding education status, 31.0% (n=13) of the respondents had primary education, 16.7% (n=7) were graduates, and a small proportion (2.4%, n=1) had postgraduation education. Notably, 7.1% (n=3) of the respondents were illiterate. The most common occupation was homemaker, accounting for 33.3% (n=14) of the respondents. This was followed by service (23.8%, n=10), business (14.3%, n=6), and student (14.3%, n=6). The demographic details of the respondents are presented in Table 1.

**Table 1:** Demographic details of the respondents

Characteristics	n	%
Sex		
Male	24	57.1
Female	18	42.9
Age (years), mean ± SD	44.38 ± 14.4	-
<40 years	12	28.6
40-49 years	10	23.8
50-59 years	13	31.0
60 years and above	7	16.7
Education status		
Primary	13	31.0
SSC	10	23.8
HSC	8	19.0
Graduation	7	16.7
Postgraduation	1	2.4
Illiterate	3	7.1
Occupation		
Farmer	2	4.8
Service	10	23.8
Homemaker	14	33.3
Business	6	14.3
Student	6	14.3
Others	4	9.5
Total	42	100.0

Clinical characteristics

Regarding laterality, 47.6% (n=20) of the respondents had surgery on their right eye, while 52.4% (n=22) had surgery on their left eye. The majority of the surgeries (57.1%, n=24) were performed due to TRD caused by advanced diabetic eye disease. The remaining 42.9% (n=18) of the surgeries were performed due to RRD. The laterality and causes of surgery among the respondents are summarized in Table 2.

**Table 2:** Laterality and causes of surgery ADED, advanced diabetic eye disease; RRD, rhegmatogenous retinal detachment; TRD, tractional retinal detachment.

Characteristics	n	%
Laterality of the eye		
Right eye	20	47.6
Left eye	22	52.4
Total	42	100.0
Causes of surgery		
RRD	18	42.9
TRD due to ADED	24	57.1
Total	42	100.0

The time frame for SOR is shown in Table 3. A significant proportion of respondents (54.8%, n=23) underwent SOR within three to six months. This was followed by 33.3% (n=14) who had SOR after six to nine months. A smaller percentage of respondents had SOR within 9 to 12 months (7.1%, n=3) or after 12 months (4.8%, n=2).

**Table 3:** Time for SOR in months SOR, silicon oil removal.

Time for SOR in months	n	%
SOR within >3 to 6 months	23	54.8
SOR after >6 to 9 months	14	33.3
SOR within >9 to 12 months	3	7.1
SOR after 12 months	2	4.8
Total	42	100.0

Visual acuity outcomes

Preoperative Visual Acuity

Preoperative visual acuity of the respondents is presented in Table 4. Using the ICD-11 (2018) classification, the majority of patients (66.7%, n=28) were classified as blind (presenting visual acuity: <3/60). Severe impairment (<6/60 to ≥3/60) was observed in 16.7% (n=7) of patients, moderate impairment (<6/18 to ≥6/60) in 11.9% (n=5), and mild impairment (<6/12 to ≥6/18) in 4.8% (n=2).

**Table 4:** Preoperative visual acuity of the respondents NLP, no light perception; PL, perception of light.

Preoperative visual acuity	n	%
Mild impairment: <6/12 to ≥6/18	2	4.8
Moderate impairment: <6/18 to ≥6/60	5	11.9
Severe impairment: <6/60 to ≥3/60	7	16.7
Blindness: <3/60 (including PL and NLP)	28	66.7
Total	42	100.0

Postoperative Visual Acuity

Postoperative visual acuity one month after surgery is detailed in Table 5. The proportion of blind patients (<3/60) decreased from 66.7% (n=28) preoperatively to 45.2% (n=19) postoperatively. McNemar's test demonstrated that this reduction was statistically significant (χ²=9.31, df=1, p=0.002). Mild impairment was achieved in 11.9% (n=5) of patients who had no mild vision preoperatively.

**Table 5:** Postoperative visual acuity of the respondents after one month *McNemar's test was statistically significant (χ²=9.31, df=1, p=0.002). NLP, no light perception; PL, perception of light.

Postoperative visual acuity	n	%	p-value
Mild impairment: <6/12 to ≥6/18	5	11.9	p=0.002*
Moderate impairment: <6/18 to ≥6/60	8	19.0	
Severe impairment: <6/60 to ≥3/60	10	23.8	
Blindness: <3/60 (including PL and NLP)	19	45.2	
Total	42	100.0	

Change in Visual Acuity

The Wilcoxon signed-rank test was used to compare preoperative and postoperative visual acuity categories. As shown in Table 6, the improvement in visual acuity was statistically significant for patients in the blindness category (Z = -3.127, p=0.002) and for the overall study population (Z = -4.128, p<0.001). Overall, 15 (35.7%) patients showed improvement in visual acuity, 23 (54.8%) remained unchanged, and 4 (9.5%) showed worsening of visual acuity. The median visual acuity category improved from blindness preoperatively to severe impairment postoperatively.

**Table 6:** Visual outcome of the respondents *The overall improvement in visual acuity from preoperative to postoperative was statistically significant (Wilcoxon signed-rank test, Z = -4.128, p<0.001).

Preoperative category	n	Improved, n (%)	Unchanged, n (%)	Worsened, n (%)	p-value
Mild impairment	2	0 (0.0)	1 (50.0)	1 (50.0)	—
Moderate impairment	5	1 (20.0)	3 (60.0)	1 (20.0)	—
Severe impairment	7	2 (28.6)	3 (42.9)	2 (28.6)	—
Blindness (<3/60)	28	12 (42.9)	16 (57.1)	0 (0.0)	—
Total	42	15 (35.7)	23 (54.8)	4 (9.5)	<0.001*

Anatomical outcomes

**Figure 1:**
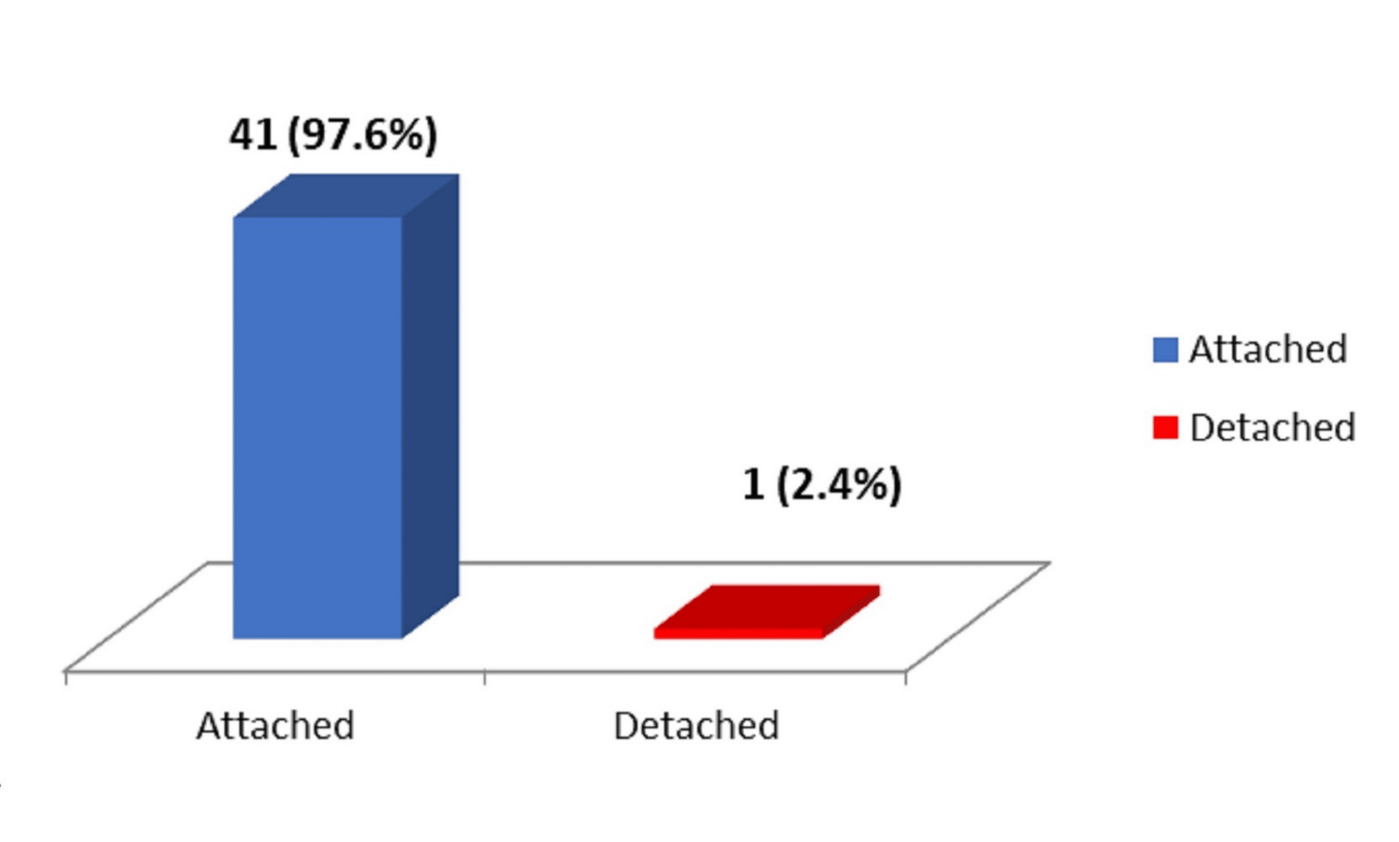
Status of the retina

The status of the retina after operation is illustrated in Figure 1. The vast majority of postoperative cases (97.6%, n=41) had an attached retina, indicating a successful surgical outcome in terms of retinal attachment. Only a small percentage (2.4%, n=1) had a detached retina after operation.

Postoperative complications

Postoperative complications are presented in Table 7. The most common complication observed was temporary IOP elevation, affecting 16.7% (n=7) of patients. Other complications included optic atrophy (11.9%, n=5), corneal edema (9.5%, n=4), mild anterior chamber exudates (4.8%, n=2), and exposed suture (2.4%, n=1). The majority of patients (54.8%, n=23) experienced no complications.

**Table 7:** Postoperative complications of the patients IOP, intraocular pressure.

Postoperative complications	n	%
Temporary IOP elevation	7	16.7
Mild anterior chamber exudates	2	4.8
Corneal edema	4	9.5
Optic atrophy	5	11.9
Exposed suture	1	2.4
None	23	54.8
Total	42	100.0

## Discussion

SO tamponade remains the preferred method for managing high-risk or complex retinal detachments, particularly those complicated by PVR, giant retinal tears, combined tractional and RRDs in diabetic retinopathy, and post-traumatic scenarios, including intraocular foreign bodies [12-14]. Extensive research confirms the efficacy of SO tamponade in reattaching the retina following severe PVR [12-14]. PPV with SO tamponade has thus become a standard technique in managing complicated RD cases.

Long-term ocular complications associated with SO include cataract formation, glaucoma, and band keratopathy. The risk of developing cataracts and glaucoma increases with the duration of SO tamponade. In some cases, even a brief period of SO tamponade can lead to cataract formation. Consequently, SO is considered a temporary tamponade agent, and its removal is recommended after achieving successful retinal reattachment to prevent potentially sight-threatening complications [14-21].

Cataract surgery is frequently necessary at the time of SOR to ensure safe removal. Combining SOR with cataract surgery generally results in better visual outcomes than separate procedures [22,23]. However, this combined approach presents challenges such as difficulties in calculating IOL power and potential corneal damage from SO contact with the corneal endothelium. Additionally, dislocation of the IOL can occur if the posterior capsulorhexis is too large. Various methods for combined SOR and cataract surgery have been described, differing primarily in the technique used for SOR. In this study, all SOR procedures were performed using a trans-scleral approach.

This prospective study aimed to evaluate the early postoperative outcomes, including anatomical and visual outcomes, along with associated complications. Patients with other retinal pathologies were excluded. The study cohort included 42 patients, with the majority (31.0%) aged 50-59 years, followed by 23.8% aged 40-49 years. The mean age was 44.38 ± 14.4 years, ranging from 16 to 67 years. There were 24 (57.1%) male and 18 (42.9%) female patients. This contrasts with a study by Haseeb et al. [24], which reported a mean age of 52.35 ± 9.8 years and a gender distribution of 66.66% male and 33.33% female. Karimi et al. [25] also reported a mean age of 51.45 ± 11.59 years. Variations in age and gender distribution across studies may be attributed to demographic and geographic differences.

Regarding preoperative visual acuity, the majority of patients (66.7%) were classified as blind (presenting visual acuity: <3/60). The second-largest group was classified as "severe vision impairment," representing 16.7% of patients. Tangpontirak et al. [26] identified predictors of visual outcomes as good best-corrected visual acuity before SOR and pseudophakia status, while predictors of poor visual outcomes included circumferential peripheral retinopexy and additional endo laser during SOR. The inability to identify all preoperative, peroperative, and postoperative predictors of surgical success in this study is likely due to the small subgroup sample sizes. Larger prospective studies are needed to identify significant predictors.

The optimal timing for SOR remains debated, with recommendations ranging from three to six months of sustained retinal attachment [19,27]. In this study, the duration of oil tamponade did not significantly affect anatomical outcomes, consistent with other reports [28-30]. However, some studies, such as Lam et al. [31], found a higher rate of redetachment with shorter tamponade durations. In our study, 54.8% of patients underwent SOR within three to six months, and 33.3% had SOR after six to nine months.

Postoperative visual acuity improved in 35.7% of patients. Similar results were reported by Haseeb et al. [24], Antoun et al. [32], and Ramezani et al. [33], who found better visual outcomes or maintained visual acuity at follow-up. Al-Habboubi et al. [23] noted functional vision restoration in approximately one-third of cases. Given that our study included highly compromised eyes with extensive posterior segment pathology, achieving visual improvement in over one-third of cases (34.69%) is considered a favorable outcome. The study also highlighted the need for further investigation into diabetic retinopathy, a common cause of TRDs and vitreous hemorrhages.

The retinal redetachment rate following SOR in this study was 2.4%, lower than the rates reported in other studies, which range from 6% to 34% [34-43]. For example, Nagpal et al. [30] reported a detachment rate of 12.7%, while Jain et al. [29] noted 11.6%. Mancino et al. [44] and Choudhary et al. [45] reported redetachment rates of 20.2% and 27.6%, respectively. The lower rate in our study may be attributed to the smaller sample size and shorter follow-up duration.

Complications following SOR were also analyzed. Ocular hypertension was the most common complication, consistent with findings by Tangpontirak et al. [26], and temporary IOP elevation was observed in 16.7% of patients. While early SOR theoretically reduces complications and improves visual outcomes, timing did not significantly impact final visual outcomes in this study.

This study has several limitations that should be considered when interpreting the results. First and most importantly, the minimum follow-up of one month is short. Many clinically relevant complications after SOR -- including late retinal redetachment, recurrent PVR, cystoid macular edema, secondary glaucoma, refractive surprises, IOL decentration, and posterior capsule opacification -- often manifest beyond the first postoperative month. Published studies evaluating SOR outcomes typically report follow-up of 3 to 12 months or longer. The sample size of 42 eyes limits statistical power for subgroup analyses and detection of rare complications. The absence of a control group (e.g., staged phacoemulsification after prior SOR) precludes direct comparison of combined versus sequential surgery. Important prognostic variables were not available in the clinical records, including duration of retinal detachment, macular status (on/off), PVR grade, SO viscosity, number of previous retinal surgeries, extent of endolaser or retinectomy, preoperative IOP, diabetic retinopathy severity grading, and formal cataract density assessment. Visual acuity was assessed with Snellen charts rather than Early Treatment of Diabetic Retinopathy Study (ETDRS) charts, and conversion to the logarithm of the minimum angle of resolution (LogMAR) with line-gain analysis was not performed in the primary analysis. The study was conducted at a single tertiary center in Bangladesh; generalizability to other settings may be limited. Finally, purposive sampling and the single-surgeon design, while appropriate for this focused clinical question, introduce potential selection and performance bias. Despite these limitations, the prospective design, standardized surgical technique, and clear inclusion criteria provide valuable real-world data on early outcomes of a practical combined procedure.

## Conclusions

Combined phacoemulsification with concurrent SOR demonstrated favorable early anatomical outcomes, with retinal attachment in 97.6% of cases and modest functional visual improvement (35.7% of patients showing category improvement) in selected eyes with previous retinal detachment repair. The procedure offers the practical advantage of eliminating the need for a separate cataract extraction surgery. However, given the short (one-month) follow-up, these findings should be interpreted as early postoperative results. Longer-term studies with extended follow-up are required to assess the durability of retinal attachment, late complications, and sustained visual recovery.
